# Plasma Hsa-miR-92a-3p in correlation with lipocalin-2 is associated with sepsis-induced coagulopathy

**DOI:** 10.1186/s12879-020-4853-y

**Published:** 2020-02-19

**Authors:** Yishan Wang, Huijuan Wang, Chunfang Zhang, Chao Zhang, Huqin Yang, Ruiyue Gao, Zhaohui Tong

**Affiliations:** 1 Department of Respiratory and Critical Care Medicine, Beijing Engineering Research Center of Respiratory and Critical Care Medicine, Beijing Chao-Yang Hospital, Capital Medical University, Beijing Institute of Respiratory Medicine, NO. 8, Gong Ti South Road, Chao-Yang District, Beijing, 100020 China; 2 Department of Anesthesiology, Pain Medicine and Critical Care Medicine, Aviation General Hospital of China Medical University and Beijing Institute of Translational Medicine, Chinese Academy of Sciences, Beijing, 100012 China

**Keywords:** Sepsis, Coagulation disorder, microRNA, Biomarker, Diagnosis

## Abstract

**Background:**

Sepsis is a life-threatening situation, and it can be rendered more severe by coagulopathy. We here examine a novel plasma biomarker for sepsis-induced coagulopathy.

**Methods:**

A total of 116 patients diagnosed with sepsis were recruited and divided into two groups by whether they also had coagulopathy. Plasma samples were collected on arrival at the intensive care unit. Fifteen sepsis-alone and 15 sepsis-induced coagulopathy plasma samples were mixed and sent for microRNA sequencing. Differently expressed microRNAs were then validated by quantitative reverse transcriptase polymerase chain reaction in 52 sepsis-alone and 34 sepsis-induced coagulopathy patients; plasma lipocalin-2 was measured as well.

**Results:**

Four microRNAs were selected from microRNA sequencing. Only hsa-mir-92a-3p was differently expressed in the validation set. Its level of expression was significantly lower in sepsis-induced coagulopathy group. Hsa-mir-92a-3p had an area under a receiver operating characteristic curve of 0.660 (95% confidence interval, 0.537, 0.782). The plasma Hsa-mir-92a-3p level was related to activated partial thromboplastin time, prothrombin activity, and plasma lipocalin-2 level. A binary logistic model showed an association between hsa-mir-92a-3p and fibrinogen with SIC.

**Conclusions:**

The utility of hsa-mir-92a-3p as a biomarker for sepsis-induced coagulopathy needs more verification, and the regulatory mechanism of hsa-mir-92a-3p in coagulation disorder and its potency as a therapeutic target must be confirmed.

## Background

Sepsis is a series of life-threatening organ dysfunction events initiated by dysregulated host response to infection [1]. Disseminated intravascular coagulation (DIC) is a major cause of organ dysfunction in sepsis. It is significantly closely related to mortality and difficult to be recognized before coagulation collapsing [2, 3]. Recently, multiple studies have confirmed that early diagnosis and intervention of DIC is crucial to disease prognosis [4–9], which highlighted the importance of identifying sepsis-induced coagulopathy (SIC) before it is too late.

MicroRNA (miRNA) is a group of endogenous noncoding small RNAs. They are highly conserved and function as posttranscriptional regulators of gene expression [10]. It has been reported that levels of miRNAs are stable across the human circulatory system [11, 12]. MiRNAs have been studied widely as biomarkers for several diseases. MiR-146a, miR-150, miR-4772-5p, miR-15a, miR-223, and miR-499-5p are potential biomarkers for sepsis in human serum/plasma, and down-regulation of miR-574-5p has been shown to be associated with higher risk of mortality [13–18]. However, there has been little study of miRNAs for SIC, according to Wang et al. Some miRNAs have been found to be related to sepsis prognosis. Among these, serum miR-122 was found to be related to coagulation disorder in sepsis patients. Thus, studies on miRNAs in association with SIC are sorely needed.

Lipocalin-2, also called neutrophil gelatinase-associated lipocalin (NGAL), is a 25 kD protein secreted by human neutrophils. Lipocalin-2 is widely used as an index in clinical settings. It can be detected in serum and plasma samples easily and quickly in many inflammatory and metabolic disorders. Its relationship with sepsis has been studied extensively. It has been used as a biomarker for sepsis-associated acute kidney injury and severe sepsis prognosis [19]. One recent study revealed its regulatory role in gut-origin sepsis [20]. Another study indicated that plasma lipocalin-2 levels are elevated in DIC patients and related to disease prognosis [21]. Though the relationship of lipocalin-2 with sepsis and DIC has been studied, no study has yet evaluated its relationship with SIC.

In this study, we sequenced plasma microRNA in sepsis-alone and SIC patients. Four microRNAs were found to be differently expressed including hsa-mir-92a-3p. In the subsequent PCR validation, we observed the hsa-mir-92a-3p expression level was consistent with sequencing results and was associated with plasma lipocalin-2 levels. We then explored the potential of hsa-mir-92a and lipocalin-2 as disease-specific markers.

## Methods

### Study design and patient characteristics

Patients were diagnosed with sepsis according to “The Third International Consensus Definitions for Sepsis and Septic Shock (Sepsis-3)” [1]. Exclusion criteria were as follows: ① younger than 18 years old, ② immunocompromised status, ③ pregnancy, ④ malignancy, ⑤ massive transfusion within 24 h of sample collection.

A total of 116 patients (49 SIC and 67 sepsis-alone) were divided into two groups: SIC group and sepsis-alone (SA) group. SIC was defined as proposed by Iba et al. in 2017 [22]: SIC scores no less than 4 points with total score of prothrombin time and coagulation exceeding 2 points. The SIC score system (Table 1) consisted of three parts: Prothrombin time, which was assessed using PT-INR value; coagulation, assessed by platelet count; and total SOFA, calculated using 4 items (respiratory SOFA, cardiovascular SOFA, hepatic SOFA, and renal SOFA). The SIC score system was proved to be closely correlated with mortality rate. It took the SOFA score into consideration, which was tailored for sepsis-associated coagulopathy [22–24].

**Table 1:** Score system for sepsis-induced coagulopathy

Category	Parameter	0	1	2
Prothrombin time	PT-INR	≤1.2	> 1.2	> 1.4
Coagulation	Platelet count (× 10^9^/L)	≥150	< 150	< 100
Total SOFA	SOFA four items	0	1	≥2

*SOFA* Sequencing Organ Failure Assessment

### Blood sample and plasma MiRNA isolation

Blood samples were collected from all patients within 24 h of admission to the intensive care unit (ICU) and centrifuged at 3000 rpm for 15 min at room temperature. The supernatant was transferred to Eppendorf tubes and stored at − 80 °C until miRNA extraction. Total plasma miRNA was isolated using a serum/plasma miRNA isolation and extraction kit (Tiangen, Beijing, China) according to the manufacturer’s instructions.

### Hiseq sequencing and target MiRNA selection

The plasma samples of 15 SIC patients and 15 SA patients were pooled in two separate groups. Total miRNA were isolated using miRcute miRNA extraction and isolation kit (Tiangen, Beijing, China); gene sequencing was completed by the Beijing Genomics Institute (BGI, Beijing, China).

MiRNAs selected for further validating all met the following two criteria (1) over 20 read numbers; (2) log2Ratio ≥ 2 [25, 26].

### Target MiRNA validation via qRT-PCR

CDNA was synthesized using a miRcute Enhanced miRNA cDNA First-line Synthesis Kit (kr211) and real-time qPCR was performed with a SYBR Green miRNA assay (Tiangen, Beijing, China). Briefly, 8 μl of isolated miRNA was added to a 20 μl system, 42 °C for 60 min followed by 95 °C 3 min for reverse transcription. Two μl of cDNA was added to a 20-μl system tested with ABI 7500 Real-time PCR System in duplication. The program was set according to the manufacture’s instruction.

### Plasma Lipocalin-2 assessment

Lipocalin-2 levels were measured using an enzyme-linked immunosorbent assay (ELISA) kit (Life Technologies, CA, US) with a range of 7.81 to 500 pg/ml. Concentration was determined from standard curves. ELISA was performed in duplicate according to the manufacturer’s instructions.

### Statistical analysis

Expression levels of selected miRNAs detected by qRT-PCR are here presented with raw Ct values. Results for normally distributed continuous variables were given as means±standard errors and compared between groups using Student’s *t* tests. Results for non-normally distributed continuous variables are summarized as medians (interquartile ranges) and compared using the Mann-Whitney U tests. Pearson’s analysis was used to evaluate the relationships between pairs of variables. Statistical significance was set at *P* < 0.05. SPSS 20.0 software was used for all statistical analyses.

A binary logistic regression model was generated after the following hypothesis was tested: ① There is a linear relationship between the continuous independent variable and the logit conversion value of the dependent variable; ② There is no multicollinearity between independent variables; ③ There are no obvious outliers, leverage points, or strong influence points. The variables that showed statistically significant differences in univariate analysis were entered into the binary logistic regression model. Variables were excluded for existence of multicollinearity between independent variables. Finally, six variables were taken into the final analysis as shown in the results.

## Results

### Patients

A total of 116 sepsis patients were included between Nov 2014 and Aug 2017 from emergency intensive care units, respiratory intensive care units, and surgery intensive care units of Beijing Chao-Yang Hospital. Eighty-six patients were included for the validation set and were divided into SIC group (*n* = 34) and SA group (*n* = 52). Patients’ clinical characteristics are presented in Table 2. Patients from the two groups were matched by sex (*P* = 0.983), age (*P* = 0.316) and disease severity measured by Acute Physiology and Chronic Health Evaluation (APACHE) II scores (*P* = 0.635). Sources of infection causing sepsis included pulmonary infection, biliary and pancreatic infection, peritonitis, urinary infection, and cerebral infection. There were significantly more cases of pulmonary infection (*P* < 0.001) and peritonitis (*P* < 0.001) in the SIC group than in the SA group. Patients were matched for other causes: biliary system and pancreatic infection (*P* = 0.276), urinary infection (*P* = 0.380), and cerebral infection (*P* = 1.000). Most global coagulation tests differed significantly between the two groups except D-dimer (*P* = 0.097) and thrombin time (TT) (*P* = 0.101). There was no difference between the two groups with respect to creatinine (*P* = 0.575) or blood urea nitrogen (BUN) (*P* = 0.299) levels.

**Table 2:** Patients’ clinical characteristics for validation set (*n* = 86)

Variables	Sepsis-induced coagulopathy (*n* = 34)	Sepsis alone (*n* = 52)	*P*-value
Sex, F/M	61.90%	62.50%	*P* = 0.983^a^
Age, years	59.56 ± 17.131	63.48 ± 17.927	*P* = 0.316^b^
Source of infection%			
Pulmonary	58.00% (29)	42.00% (21)	*P* < 0.001^a^
Biliary system and pancreatic	45.50% (5)	54.50% (6)	*P* = 0.276^a^
Peritonitis	78.90% (15)	21.10% (4)	*P* < 0.001^a^
Urinary system	60.00% (3)	40.00% (2)	*P* = 0.380^a^
Cerebral	0% (0)	100% (1)	*P* = 1.000^a^
SOFA scores	6.68 ± 3.67	6.04 ± 3.16	*P* = 0.393^b^
APACHE II scores	17.35 ± 7.88	16.60 ± 6.73	*P* = 0.635^b^
28-day mortality%	67.7% (21)	32.3% (10)	*P* < 0.001^a^
PLT (×10^9/L)	108.03 ± 91.99	211.10 ± 96.93	*P* < 0.001^b^
D-dimer (mg/dL)	7.12 (3.00, 18.24)	4.60 (2.38, 8.64)	*P* < 0.001^c^
APTT (s)	44.80 (38.10, 61.10)	35.75 (31.70, 43.23)	*P* < .001^c^
PT (s)	14.90 (13.48, 17.33)	12.75 (11.53, 13.88)	*P* < 0.001^c^
PA%	66.53 ± 17.89	81.10 ± 11.98	*P* < 0.001^c^
INR	1.26 (1.16, 1.47)	1.11 (1.03, 1.18)	*P* < 0.001^c^
Fbg (mg/dL)	275.13 ± 136.45	421.50 ± 173.30	*P* < 0.001^b^
TT (s)	20.00 (18.28, 24.88)	19.10 (17.98, 20.70)	*P* = 0.055^c^
Cr (mmol/L)	75.65 (52.90, 137.20)	74.25 (53.80, 127.65)	*P* = 0.575^c^
BUN (mmol/L)	10.12 (5.78, 14.07)	8.54 (5.06, 12.78)	*P* = 0.299^c^

^a^χ2 test, ^b^Student’s t test, ^c^Mann-Whitney U test, *SOFA* Sequential organ failure assessment, *APACHE II* Acute physiology and chronic health evaluation, *PLT* Platelet, *APTT* activated partial thromboplastin time, *PT* Prothrombin time, *PA* Prothrombin activity, *INR* International normalized ratio, *Fbg* Fibrinogen, *TT* Thrombin Time, *Cr* Creatine, *BUN* Blood urea nitrogen

Another 30 patients (15 SIC patients and 15 SA patients) were included for gene sequencing, demographic variables were presented in Table 3.

**Table 3:** Patients’ clinical characteristics for gene sequencing (*n* = 30)

Variables	Sepsis-induced coagulopathy (*n* = 15)	Sepsis alone (*n* = 15)	*P*-value
Sex, F/M	4/11	8/7	*P* = 0.136^a^
Age, years	62.60 ± 13.91	65.13 ± 20.40	*P* = 0.694^b^
Cause of sepsis%			
Pulmonary infection	75% (9)	25% (3)	*P* = 0.025^a^
Biliary system and pancreatic	20% (1)	80% (4)	*P* = 0.330^a^
Peritonitis	40% (4)	60% (6)	*P* = 0.439^a^
Urinary system	50% (1)	50% (1)	*P* = 1.000^a^
Cerebral	0% (0)	100% (1)	*P* = 1.000^a^
SOFA scores	9.00 ± 4.33	6.21 ± 3.39	*P* = 0.060^b^
APACHE II scores	20.20 ± 8.19	15.59 ± 7.77	*P* = 0.125^b^
PLT (×10^9/L)	131.20 ± 87.78	156.87 ± 76.27	*P* = 0.400^b^
D-dimer (mg/dL)	13.94 ± 12.41	5.72 ± 5.49	*P* = 0.026^b^
APTT (s)	50.80 (39.70, 60.70)	34.90 (33.40, 53.20)	*P* = 0.062^c^
PT (s)	17.00 (14.20, 20.10)	12.90 (12.50, 18.00)	*P* = 0.022^c^
PA%	62.15 ± 19.45	70.51 ± 14.57	*P* = 0.193^b^
INR	1.51 ± 0.54	1.30 ± 0.29	*P* = 0.197^b^
Fbg (mg/dL)	294.60 (140.60, 340.80)	310.30 (254.50, 533.40)	*P* = 0.262^c^
TT (s)	21.50 (19.20, 114.45)	23.00 (18.70, 282.90)	*P* = 0.787^c^
Cr (mmol/L)	137.40 (74.05, 232.75)	129.56 (77.80, 301.15)	*P* = 0.967^c^
BUN (mmol/L)	11.32 ± 6.49	11.45 ± 6.38	*P* = 0.959^b^

^a^χ2 test, ^b^Student’s t test, ^c^Mann-Whitney U test, *SOFA* Sequential organ failure assessment, *APACHE II* Acute physiology and chronic health evaluation, *PLT* Platelet, *APTT* Activated partial thromboplastin time, *PT* Prothrombin time, *PA* Prothrombin activity, *INR* International normalized ratio, *Fbg* Fibrinogen, *TT* Thrombin Time, *Cr* Creatine, *BUN* Blood urea nitrogen

### Hsa-mir-92a-3p expressed differently between SIC group and SA group

**Fig. 1:**
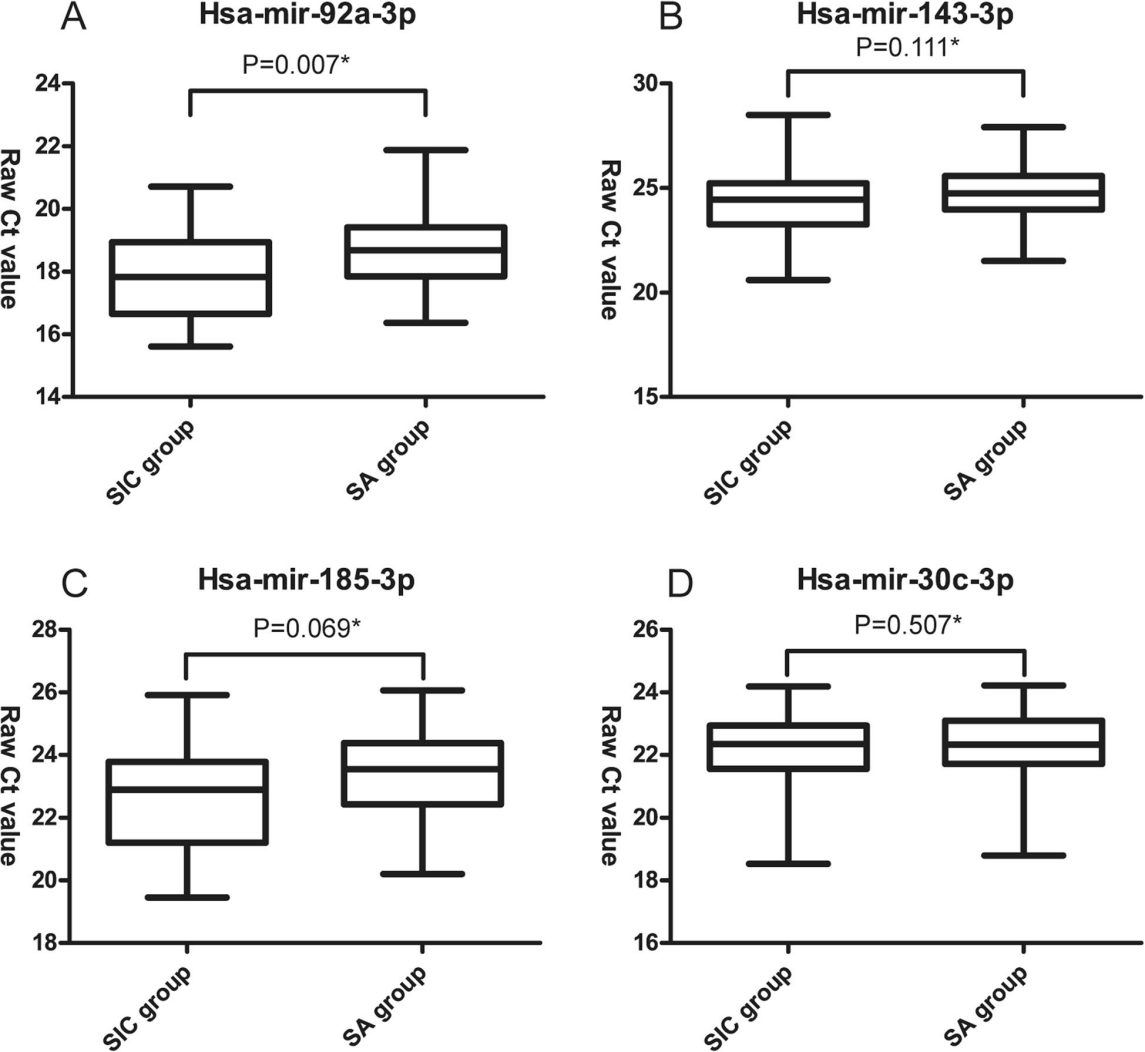
Comparisons of the levels of four miRNAs between SIC group (*n* = 34) and SA group (*n* = 52). **a**. Comparison of plasma hsa-mir-92a-3p between SIC and SA group, **b**. Comparison of plasma hsa-mir-143-3p between SIC and SA group, **c**. Comparison of plasma hsa-mir-185-3p between SIC and SA group, **d**. Comparison of plasma hsa-mir-30c-3p between SIC and SA group, * by Student’s t test, SIC = Sepsis-Induced Coagulopathy, SA = Sepsis-Along

According to the results of gene sequencing, we chose hsa-miR-143-3p, hsa-miR-185-3p, hsa-miR-92a-3p, and hsa-miR-30c-3p for further measurement; mean reads and log2 ratio of four miRNAs are shown in Table 4. The Student’s *t* test revealed significance only for level of hsa-mir-92a-3p: SIC group vs. SA group (17.86 ± 1.44 vs. 18.66 ± 1.21, *P* = 0.007) (Fig. 1).

**Table 4:** Differently expressed miRNAs in miRNAs sequencing

miR_name	SA Reads	SIC Reads	log2Ratio	*P*-value	q-value
hsa-miR-92a-3p	9184	9651	−4.96	0	0
hsa-miR-143-3p	5959	9641	−4.33	0	0
hsa-miR-30c-5p	645	335	−5.97	0	0
hsa-miR-185-5p	663	2718	−2.99	0	0

*miR* microRNA, *SA* Sepsis Along, *SIC* Sepsis Induced Coagulopathy

### Plasma Hsa-mir-92a level related to global coagulation index

We recorded patients’ global coagulation tests results including D-dimer, activated partial thromboplastin time (APTT), prothrombin activity (PA), fibrinogen (FBG), and thrombin time (TT). Statistical analysis revealed a strong relationship between hsa-mir-92a and PA (Pearson’s correlation coefficients = 0.266, *P* = 0.013) and hsa-mir-92a and APTT (Pearson’s correlation coefficients = − 0.325, *P* = 0.002).

### Plasma Hsa-miR-92a level related to plasma Lipocalin-2 level

**Fig. 2:**
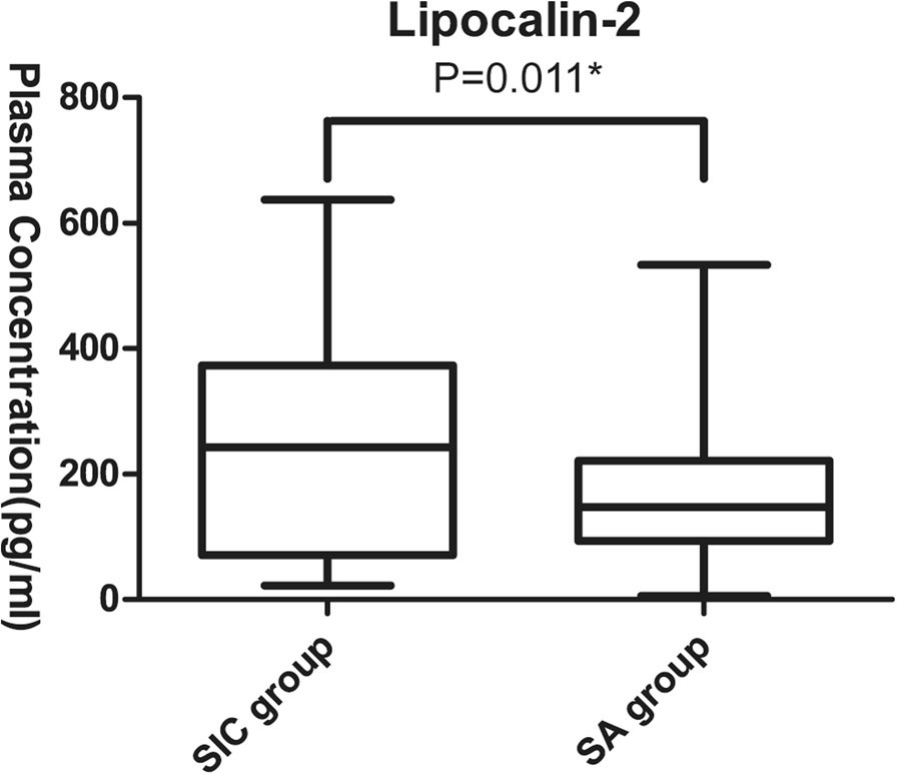
Comparisons of the levels of lipocalin-2 between SIC group (*n* = 34) and SA group (*n* = 52). * Student’s t test, SIC = Sepsis-Induced Coagulopathy, SA = Sepsis-Along

We measured plasma lipocalin-2 levels and found plasma lipocalin-2 expression to differ between the SIC and SA groups (246.05 ± 169.95 pg/ml vs. 168.84 ± 105.30 pg/ml, *P* = 0.011) (Fig. 2) and to be related to plasma hsa-miR-92a level (Pearson’s correlation coefficients = − 0.282, *P* = 0.009).

### Diagnostic values of Hsa-miR-92a and Lipocalin-2

**Fig. 3:**
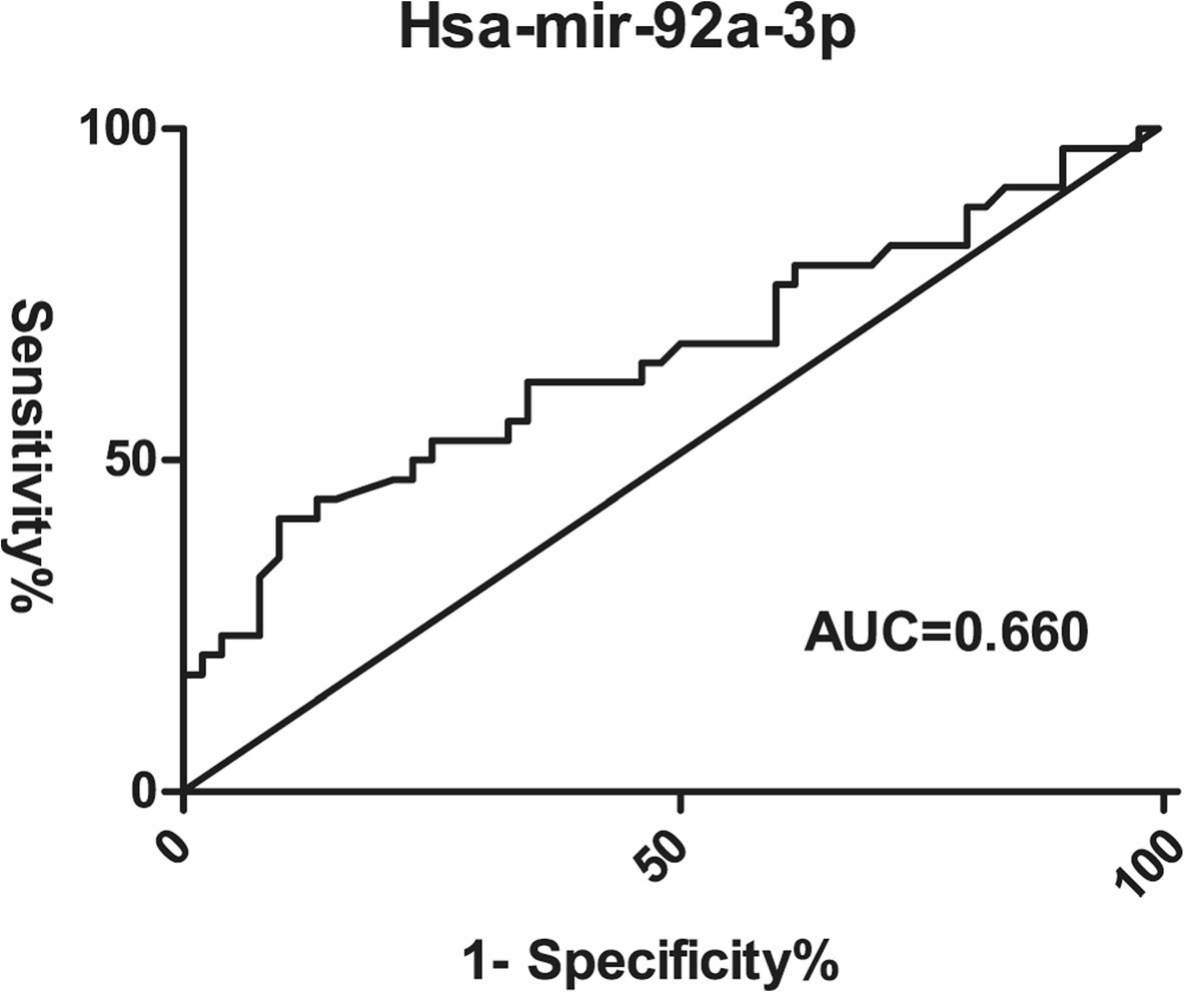
Receiver operating characteristic curves of hsa-mir-92a-3p for SIC group (*n* = 34) and SA group (*n* = 52). AUC = Area Under Curve

**Fig. 4:**
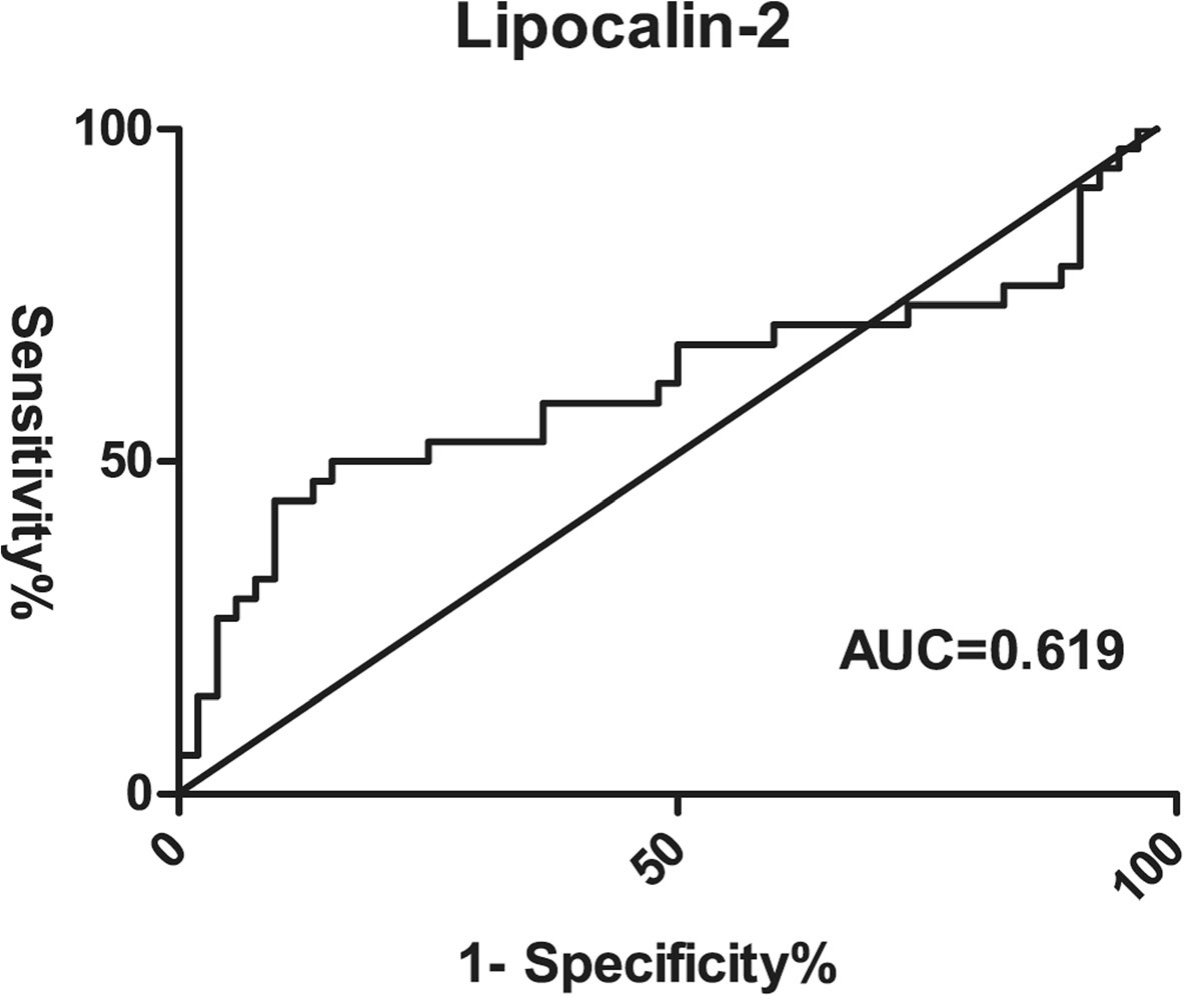
Receiver operating characteristic curves of lipocalin-2 for SIC group (*n* = 34) and SA group (*n* = 52). AUC = Area Under Curve

Results showed hsa-miR-92a and lipocalin-2 could be used to distinguish sepsis-induced coagulopathy from sepsis-alone patients. To compare the diagnostic values of these two biomarkers, receiver operating characteristic (ROC) curves were generated and area under the curve (AUC) was calculated. Hsa-miR-92a had an AUC of 0.660 (*P* = 0.011, 95% CI, 0.537, 0.782) in predicting the absence of SIC whereas lipocalin-2 had an AUC of 0.619 (*P* = 0.084, 95% CI, 0.485, 0.752) in predicting SIC (Fig. 3, Fig. 4). When the cutoff point was set at 261.01 pg/mL, lipocalin-2 had the highest specificity of 84.6% (Table 5).

**Table 5:** Diagnostic values of has-miR-92a and lipocalin-2

Biomarker	AUC (95% CI)	Cutoff point	Sensitivity%	Specificity%
Hsa-miR-92a	0.660 (0.537, 0.782)	17.18	41.20%	58.80%
lipocalin-2	0.619 (0.485, 0.752)	261.01 pg/mL	50%	84.60%
Hsa-miR-92a + lipocalin-2	0.687 (0.578, 0.782)	0.46139	52.94%	84.62%

*AUC* Area under curve

**Fig. 5:**
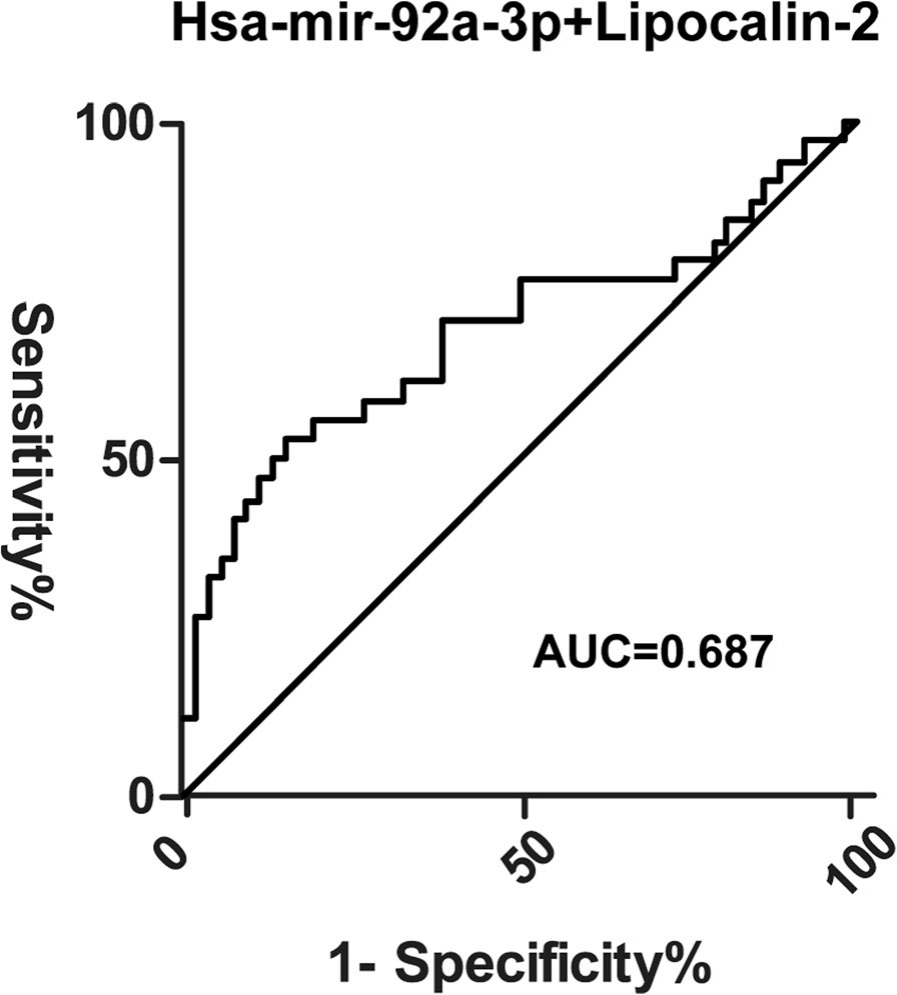
Receiver operating characteristic curves of hsa-mir-92a-3p and lipocalin-2 for SIC group (*n* = 34) and SA group (*n* = 52). AUC = Area Under Curve

We then generated the ROC for a combination of hsa-mir-92a and lipocalin-2 (Fig. 5). The AUC for hsa-mir-92a + lipocalin-2 was 0.687 (*P* = 0.003, 95% CI, 0.578, 0.782).

### Risk factors for Sepsis-induced coagulopathy

As shown, hsa-mir-92a was differently expressed between SIC and SA groups. Because we hypothesized sepsis accompanied by coagulation disorder indicated more disease severity, APACHEII score was also taken into analysis. The analysis of basic information indicated a difference in the number of patients with each specific source of pulmonary infection and peritonitis; these variables were taken into a binary logistic regression model.

Finally, we used a binary logistic regression model to assess the influence of the plasma level of hsa-mir-92a, APACHEII score, pulmonary infection and peritonitis, D-dimer level, and fibrinogen for SIC. There were linear relationships between dependent variables and covariates and one case with a ZResid of 3.386 times the standard deviation. These were analyzed further. The model was significant, 0.531 for Hosmer and Lemeshow goodness of fit test. Among the six independent variables included in the model, mir-92a and fibrinogen had statistical significance. For each unit of increase in plasma mir-92a level, the risk of SIC decreased by 32.4%, and for each unit of increase in plasma fibrinogen level, the risk of SIC decreased by 0.7%. The result and odds ratio with its 95% CI are given in Table 6.

**Table 6:** Risk factors for sepsis-induced coagulopathy

				95% CI	
Variables	DF	*P* value	OR	Lower	Upper
Hsa-mir-92a	1	0.035	0.658	0.446	0.970
Fibrinogen	1	0.001	0.993	0.989	0.997
D-dimer	1	0.093	1.050	0.992	1.111
APACHEII	1	0.937	1.003	0.932	1.080
Pulmonary Infection	1	0.402	0.600	0.182	1.979
Peritonitis	1	0.857	0.853	0.150	4.856
Constant	1	0.019	10,504.227		

The binary logistic model included six variables: plasma level of hsa-mir-92a, Fibrinogen, D-dimer, APACHE II score, pulmonary infection and Peritonitis. *APACHEII* Acute physiology and chronic health evaluation, *DF* Degree of freedom, *OR* Odds ratio, *CI* Confident interval

## Discussion

Sepsis is a life-threatening situation. It is often accompanied by coagulation disorder, and it can leave clinicians stymied in the attempt to identify the optimum treatment. It would be useful to find new biomarkers and therapeutic targets. In this study, we found novel biomarkers for SIC. After Hiseq sequencing, we located four differently expressed miRNAs in human plasma and validated our findings in a larger sample. Plasma lipocalin-2 was measured as well. We found significant differences in both hsa-mir-92a and lipocalin-2 levels between SIC and SA groups. There were higher plasma levels of hsa-mir-92a in the SA group than in the SIC group, but the higher plasma lipocalin-2 level indicated a greater possibility of SIC. We also found a strong relationship between hsa-mir-92a and prothrombin activity, activated partial thromboplastin time, and lipocalin-2.

MiR-92a has been widely studied in tumorigenesis and metastasis of several different kinds of tumors. MiR-92a is significantly up-regulated in colon rectal cancer tissues and correlated with metastases and poor prognosis [27]. It also participates in gene regulation processes in other kinds of tumors including non-Hodgkin’s lymphoma, acute lymphoid leukemia, multiple myeloma, breast cancer, lung cancers, hepatocellular carcinoma, and esophageal squamous cell carcinoma, acting as both tumor suppressor and oncogene [28–34].

Mir-92a was found to be involved in angiogenesis and endothelial cell function [35]. Bonauer et al. found that human endothelial cells (ECs) express the miR-17–92 cluster, particularly its member miR-92a. Forced overexpression of miR-92a in human ECs impairs EC function, and inhibition of miR-92a can enhance vessel growth. One in vivo study with a mouse hind limb ischemia model revealed that the number of capillaries and smooth muscle actin-positive arterioles increases after antagomir-92a treatment. In searching for possible pathways and putative target genes for mir-92a, the mRNAs for integrin subunits ɑ5 (ITGA5) and ɑv, sphingosine-1-phosphate receptor 1 (S1PR1), and mitogen-activated kinase 4 (MKK4) were found to show less expression in response to miR-92a overexpression. Further study on downstream pathways by silencing ITGA5 expression, resembling the effect of miR-92a on mRNA expression, revealed the expression levels of a second group of genes might be secondarily regulated as a consequence of ITGA5 downregulation. These genes include CD31, whose product is a platelet/endothelial cell adhesion molecule, and VWF, whose product is a Von Willebrand factor. The mir-92a-ITGA5-VWF axis could lead to low expression of Von Willebrand factor and cause instability of FVIII, finally resulting in prolonged APTT.

Lipocalin-2 has been studied as a clinical biomarker for DIC in its different forms: plasma free and total lipocalin-2 levels were significantly higher in patients with overt-DIC than in those without overt-DIC. The free lipocalin-2 level also showed a significant prognostic value in DIC [36]. In the search for a possible regulatory mechanism for mir-92a and lipocalin-2, studies have focused on apoptotic cell-derived sphingosine-1-phosphate (S1P), which induces the production and release of lipocalin-2 by human macrophages through the S1P-S1PR1-signal transducer and activator of transcription 3 (STAT3)-lipocalin-2 axis in lymphangiogenesis and tumor metastasis [37, 38]. Mir-92a has been confirmed to play a role in the regulation of S1PR1 [35]. Increased mir-92a could lead to downregulation of S1PR1 and upregulation of lipocalin-2. However, this model still needs confirmation.

There are limitations to this study. First, without a basal level of miRNA expression in healthy controls, we could not determine whether the differently expressed miRNAs also differed between the SIC group and healthy controls in the same way. Second, because no optimum normalizer exists for such critical conditions as sepsis and severe infection [39], raw Ct value was taken into further analysis, which can cause bias due to individual difference and different sample degeneration states.

## Conclusions

In this work, we evaluated differently expressed miRNAs between SA and SIC patients. After validation, plasma hsa-mir-92a was confirmed as a diagnostic biomarker for sepsis-induced coagulopathy and related to global coagulation index and plasma lipocalin-2 level. Further study is needed to confirm the regulatory role of hsa-mir-92a-3p in coagulation disorders and assess therapeutic potency.

## Data Availability

The datasets used and/or analysed during the current study are available from the corresponding author on reasonable request.

## Abbreviations

APACHEII: Acute Physiology and Chronic Health Evaluation
APTT: Activated Partial Thromboplastin Time
AUC: Area Under Curve
BGI: Beijing Genomics Institute
BUN: Blood Urea Nitrogen
CI: Confidence Interval
DIC: Disseminated Intravascular Coagulation
ECs: Endothelial Cells
ELISA: Enzyme-Linked Immunosorbent Assay
FBG: Fibrinogen
ICU: Intensive Care Unit
INR: International Normalized Ratio
ITGA5: Integrin Subunits ɑ5
miRNAs: MicroRNAs
MKK4: Mitogen-activated Kinase Kinase 4
NGAL: Neutrophil Gelatinase-Associated Lipocalin
PA: Prothrombin Activity
PT: Prothrombin Time
qRT-PCR: Quantitative Reverse Transcription-Polymerase Chain Reaction
ROC: Receiver Operating Characteristic
S1P: Sphingosine-1-Phosphate
S1PR1: Sphingosine-1-Phosphate Receptor 1
SA: Sepsis-Along
SIC: Sepsis-Induced Coagulopathy
SOFA: Sequential Organ Failure Assessment
STAT3: Signal Transducer and Activator of Transcription 3
TT: Thrombin Time
VWF: Von Willebrand Factor
